# Gene Expression Profiling and Phenotypic Characterization of Circulating Tumor Cells Derived from a Murine Osteosarcoma Model

**DOI:** 10.3390/cancers17071210

**Published:** 2025-04-02

**Authors:** Malte Benje, Tamara Vitacchio, Dennis Fritsche, Walter Tinganelli

**Affiliations:** GSI Helmholtzzentrum für Schwerionenforschung, 64291 Darmstadt, Germany; m.benje@gsi.de (M.B.); t.vitacchio@gsi.de (T.V.);

**Keywords:** circulating tumor cells, osteosarcoma, mRNA-seq

## Abstract

Background/Objectives: Osteosarcoma is an aggressive bone malignancy with high metastatic potential to the lungs. CTCs, as seeds of metastasis, play an important role in the spread of this cancer, and, therefore, their isolation, culture, and gene expression analysis promises valuable insights into the progression and metastatic cascade of osteosarcoma. The aim of this study was to isolate and culture CTCs from osteosarcoma-bearing mice and compare their migration, radioresistance, and gene expression with their parental cell line. Methods: CTCs from LM8-inoculated mice were isolated and cultured. The gene expression of the CTC-derived cell lines was then compared to the parental cell line. Furthermore, a Transwell assay, a clonogenic assay after irradiation, and immunohistochemical stainings were used to compare the CTC-derived cell lines with the parental cell line. Results: The CTC-derived cell lines differed significantly in gene expression from their parental cell line. 361 differentially expressed genes were identified, among which GO and KEGG analysis revealed major differences in the expression of genes related to antigen processing and presentation and extracellular matrix constituents. In addition, the CTC-derived cell lines were observed to have a higher migratory capacity and comparable radioresistance compared to the parental cell line. CD44 expression was found to be conserved in CTC-derived cell lines. Conclusions: This study provides a comparison between CTC-derived and their parental cell lines in terms of gene expression, migration, and radioresistance. Our findings allow for further research in the field of osteosarcoma CTCs and their generation. Furthermore, the identified DEGs between CTCs and their parental cell line can serve as a reference point for targeted therapies against osteosarcoma CTCs.

## Simple Summary

Osteosarcoma is a malignant bone tumor with a high potential for metastasis, especially to the lungs. Circulating tumor cells (CTCs), considered as the seeds of metastasis, play a key role in this process. In this study, we established CTC-derived cell lines from osteosarcoma-bearing mice and compared their gene expression with the parental cell line. Our findings provide insight into the behavior of cultured CTCs and possible adaptations that facilitate CTC dissemination and survival. Furthermore, our results present a list of differentially expressed genes between the parental and CTC-derived cell lines, which could be used as a basis for future research on the metastatic cascade of osteosarcoma and its generation of CTCs.

## 1. Introduction

Osteosarcoma (OS) is the most common malignant bone tumor in children and adults, characterized by a mesenchymal origin in the population of bone-forming cells [1]. Recurrence and metastases are the leading causes of mortality in OS patients, resulting in a 5-year survival rate of approximately 20% in patients with detectable metastases at diagnosis [2]. OS predominantly metastasizes to the lungs via the hematogenous route to form metastatic lesions that, if not removed, are almost always fatal [3]. Therefore, there is a great need for biomarkers that can accurately assess the risk of metastases formation, and therapies that target the spread of OS are urgently needed.

Circulating tumor cells (CTCs) play an important role in metastases since they represent cells that have shed from the primary tumor into the bloodstream [4]. If these cells survive in the circulation, they are able to colonize distant sites and spread the disease to other organs. To successfully metastasize, CTCs must leave the primary tumor, enter the bloodstream, survive in the circulation, and extravasate at a secondary site [4]. This process requires cancer cells to acquire a variety of features and adaptations, which have been investigated primarily in epithelial tumors [5,6,7], while the underlying mechanisms of CTC formation in mesenchymal malignancies, such as OS, remain poorly understood.

For example, in triple-negative breast cancer, isolated and cultured CTCs have been shown to migrate at a higher rate than primary tumor cells [8]. It has been shown that epithelial CTCs are able to evade the surveillance of the immune system through a variety of mechanisms, such as increased expression of PDL1 [9] and CD47 [10], or by acquiring a protective coating of platelets [11].

It is also known that during the metastatic cascade, the extracellular matrix (ECM) of cancer cells undergoes extensive remodeling to allow CTCs to penetrate the endothelium and survive in the bloodstream. Ting et al. (2014) showed that ECM-associated genes such as Sparc, Mgp, and Spon2 were highly expressed in pancreatic cancer CTCs [12].

While there is a lot of research on CTCs of epithelial tumors, there is limited research on CTCs of mesenchymal origin. Regarding CTCs in OS research, there are only a few publications. Wu et al. (2018) were able to show that the number of CTCs correlates with progression and response to therapy and that the EMT phenotype of these cells correlates with the presence of distant metastases [13]. Another group used hexokinase 2 expression in mice and patients to identify CTCs and to correlate their number with disease-free survival, highlighting their key role in the metastatic spread of OS [14]. Similarly, Li et al. (2019) were able to correlate the number of CTCs and their expression of MTA1 with response to therapy and Enneking staging [15]. Despite these studies on CTCs of mesenchymal origin, to our knowledge, only a few publications compare the gene expression of OS CTCs with the transcriptome of their primary tumors. Chalopin et al. (2018) showed that cultured CTCs from a murine osteosarcoma model expressed higher levels of Adam8 and Ltk compared to their parental cell line [16]. Green et al. (2020) were able to isolate CTCs from osteosarcoma patients and used single-cell RNA sequencing to identify several genes differentially expressed between CTCs and the primary tumor [17].

In this study, we isolated and cultured CTCs in vitro from a murine OS model and compared the resulting CTC-derived cell lines to the parental LM8 cell line in terms of morphological, phenotypic, and molecular characteristics. Through RNA sequencing analysis, we identified a significant cohort of differentially expressed genes (DEGS), with a focus on those related to ECM remodeling and immune interactions. Our results provide novel insights into the biology of mesenchymal CTCs and their role in OS metastasis.

## 2. Materials and Methods

### 2.1. Cell Culture and Animal Inoculation

The highly metastatic murine cell line LM8 [18] was cultured in DMEM Glutamax (Gibco, Thermo Fisher Scientific, Waltham, MA, USA), 10% FBS (Gibco), and 1% P/S Gibco) at 37 °C and 5% CO_2_. LM8 cells are derived from Dunn osteosarcoma cells (originating from C3H/He mice, purchased from Riken BioResource Center, Kyoto, Japan). The same culture conditions were used for CTC-derived cell lines.

All animal experiments were performed using 16 11/12-week-old female C3H/He mice (Janvier Labs, Le Genest-Saint-Isle, France) overall, according to German Federal Law under the approval of the Hessen Animal Ethics Committee (Project License Da17/2000 and Da17/2005). The mice were housed at GSI in a conventional animal facility (non-SPF) at 22 °C, on a 12-h light-dark cycle, with unrestricted access to water and a standard diet (Ssniff Spezialdiäten GmbH, Soest, Germany). For the inoculation of C3H mice, 10^6^ cells were harvested with trypsin and resuspended in 25 µL PBS. The solution was then injected subcutaneously into the hind legs of the animals, resulting in a growing tumor. The tumor was then allowed to grow for 28 days, after which the animals were sacrificed via an excess dose of isoflurane followed by cervical dislocation, and blood was taken via cardiac puncture. A subcutaneous model was selected over an orthotopic one due to considerations of animal welfare, more homogenous tumor growth, and improved accessibility to the tumor site.

For the LM8 replicates in the mRNA seq experiment, LM8 cells were cultured individually for at least two passages.

### 2.2. CTC Isolation and Cultivation

**Figure 1:**
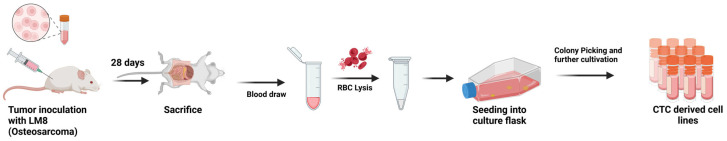
CTC isolation. Workflow of tumor injections, CTC isolation, and cultivation. Created in BioRender [20].

Red blood cells (RBCs) were lysed using the Miltenyi RBC Lysis Kit (Miltenyi Biotec, Bergisch Gladbach, Germany, Cat. No. 130-094-183) according to the manufacturer’s instructions to isolate CTCs. The remaining white blood cells (WBCs) containing CTCs were then seeded into culture flasks until colonies were formed. Some of the colonies were then picked and further cultured into cell lines (Figure 1). The isolation and cultivation were based on a previously described protocol of Tanaka et al. (2013) [19].

### 2.3. gDNA Isolation and SRY PCR

For gDNA isolation, the NucleoSpin Tissue Kit from Macherey-Nagel (Macherey-Nagel GmbH & Co., KG, Düren, Germany, Ref. 740952.50) was used according to the manufacturer’s instructions. DNA was isolated from at least 10^6^ cells. For sex determination, the following already described primers specific for the Sry gene were utilized: 5′-TGGGACTGGTGACAATTGTC-3′ and 5′-GAGTACAGGTGTGCAGCTCT-3′ [21]. The SYBR Green Master Mix (Thermo Fisher Scientific) was used with a final concentration of 0.2 µM per primer. The PCR protocol was as follows: an initial denaturation at 95 °C for 10 min, followed by 33 cycles of 95 °C for 35 s, 50 °C for 1 min, and 72 °C for 1 min. A final extension step was performed at 72 °C for 5 min. PCR products were separated on 1.5% agarose gels and visualized under UV light. As a negative control, 2 gDNA isolates from the female 4T1 cell line were used.

### 2.4. Clonogenic Survival Assays After Irradiation

Clonogenic assays after irradiation were performed as described in the protocol of Franken et al. (2006) [22]. In brief, cells were irradiated at the GSI’s in-house Seifert X-rays machine (Waygate Technologies, a Baker Hughes business, Ahrensburg, Germany) operating at 250 kV and 16 mA, with a dose rate of 2 Gy/min. Cells were then harvested, counted, and seeded at 100 cells per flask. After 10 days of incubation, colonies were stained with methylene blue, and the ones with more than 50 cells were counted. The plating efficiency was determined, and the counts were plotted according to the linear quadratic model.

### 2.5. Immunofluorescence Stainings

LM8 or CTC-derived cell lines were cultured on coverslips until they reached ~50% confluency. The cells were then fixed for 10 min with 3.7% PFA and permeabilized with 0.25% Triton X-100 (Carl Roth GmbH + Co., KG, Karlsruhe, Germany). After washing with PBS, the coverslips were blocked with 3% BSA (Sigma-Aldrich, Merck KGaA, Darmstadt, Germany) in PBS for 1 h. Subsequently, the coverslips were incubated overnight at 4 °C with the primary antibody (1:500), washed three times, and then incubated with the secondary antibody (1:1000) for 1 h. After another three washing steps, DAPI (Thermo Fisher Scientific) staining (300 ng/mL) was performed for 10 min, followed by two additional washing steps. Finally, the coverslips were mounted on microscopy slides with Permafluor (Epredia, Portsmouth, NH, USA) and stored at 4 °C. The sizes of the nuclei were measured using the thresholding and measuring function of FIJI (Version 2.16.0, ImageJ).

### 2.6. Transwell Migration Assay

For the Transwell migration assay, Millicell cell culture inserts with 8 µm pores (Merck Millipore, Merck KGaA, Darmstadt, Germany, Cat No. PTEP06H48) were used. A total of 1 mL FBS-free DMEM containing 2 × 10^5^ cells was added to the upper chamber of the Transwell insert. Then, 2 mL of DMEM containing FBS was pipetted into the wells below the inserts. The plate was incubated for 24 h. After incubation, non-migrated cells were removed using a cotton-tipped applicator. Subsequently, the Transwells were fixed in 70% EtOH for 15 min and then stained with DAPI (1 µg/mL) for 10 min. After three washing steps with PBS, the inserts were imaged with an “Echo Revolve” microscope (Discover Echo Inc., San Diego, CA, USA), and cells in at least five different areas per insert were counted.

### 2.7. RNA Isolation and mRNA Sequencing

Total RNA was isolated from cell pellets using the Machery-Nagel Nucleospin RNA Kit (Macherey-Nagel) according to the manufacturer’s instructions. The concentration and purity of RNA were assessed by NanoDrop (Colibri Microvolume Spectrometer from the Manufacturer Titertek-Berthold, Pforzheim, Germany). RNA samples were sent to Genewiz (Genewiz, Azenta Life Sciences, Burlington, MA, USA)for mRNA sequencing. Paired-end sequencing was performed with a read length of 150 bp and 20 million reads per sample after a polyA enrichment.

### 2.8. Bioinformatics

The data analysis of mRNA sequencing data was performed using the Galaxy web platform, and data analysis was done using public servers. Quality control, trimming, alignment, and DEG identification were performed as previously described [23].

Briefly, the quality of the sequencing reads was assessed using FastQC. Adaptor and poly-G reads were trimmed using Cutadapt [24] with a quality cutoff of 20 and a minimum read length of 20 bps. Mapping to the most recent mouse genome (GRCm39) was performed using RNA STAR [25]. Reads were counted using featureCounts [26], and DEGs were identified using Deseq2 [27] between the parental LM8 cell line and the CTC-derived cell lines. Heatmaps were generated with heatmap2 and show log(2 + 1) transformed z-scores. Gene ontology and KEGG analysis were performed with goseq [28]. The results of the KEGG analysis were rendered using Pathview (Galaxy Version 1.34.0+galaxy0) [29]. All other statistical analyses were performed using Graphpad Prism 10.

## 3. Results

### 3.1. Characterization of CTC-Derived Cell Lines: Confirmation of Origin and Phenotypic Properties

**Figure 2:**
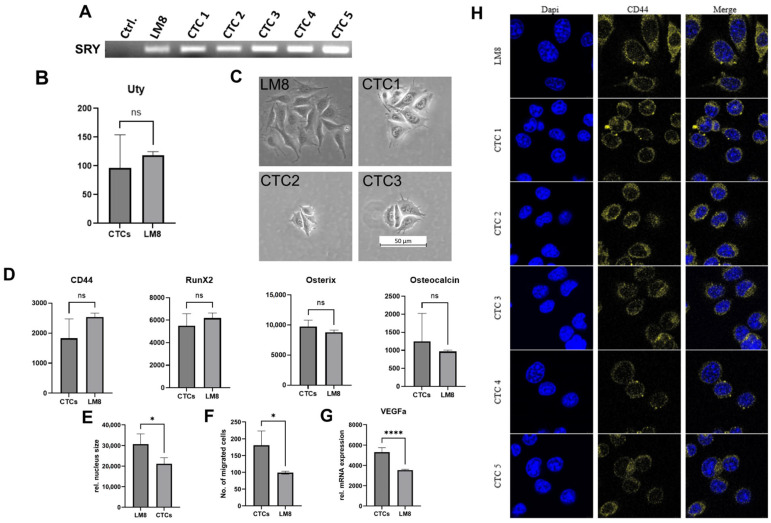
CTC isolation and in vitro characterization. (**A**) Endpoint PCR results for the Y-chromosome-specific Sry gene of LM8 and CTC-derived cell lines. (**B**) Relative mRNA expression of the Ubiquitously Transcribed Tetratricopeptide Repeat Containing, Y-Linked (Uty) gene, Wald test. (**C**) Example pictures of CTCs derived colonies and LM8. (**D**) Relative mRNA expression of OS specific markers comparing 8 CTC-derived cell lines with the original LM8 cell line, Wald-test. (**E**) The size of the nuclei was compared between LM8 and CTC derived cell lines (the means of at least 5 pictures and 2 individual stainings were analyzed), unpaired *t*-test * *p*-value < 0.05. (**F**) Number of migrated cells in a Transwell assay, LM8 was compared to 5 CTC-derived cell lines, unpaired *t*-test *, *p*-value < 0.05. (**G**) Relative mRNA expression of VEGFa in 8 CTC-derived cell lines and LM8, Wald-test, **** *p*-adj. < 0.001. (**H**) IF stainings of cultured LM8-CTCs, DAPI in blue and CD44 in gold.

Female host mice were inoculated with the LM8 cell line, and a final blood sample was taken after 28 days. Circulating tumor cells were isolated and cultured, as shown in Figure 1. Cells were grown into cell lines until sufficient material was available for DNA and RNA isolation. Since the LM8 cell line is derived from male mice, endpoint PCR for the Y chromosome-specific SRY gene confirmed the origin of the CTC-derived cell lines (Figure 2A). Gene expression analysis of the Y chromosome-specific UTY gene further confirmed their origin (Figure 2B).

In addition, we found that the filopodia formation characteristic of the LM8 cell line was retained in the CTCs (Figure 2C), as well as the expression of OS-specific markers such as RunX2, Osterix, and Osteocalcin (Figure 2D). In IF stainings, we also found that the CTC-derived cell lines had significantly smaller nuclei than the parental LM8 cell line (Figure 2E). As this feature might facilitate migration, we performed a Transwell migration assay and found that the CTC-derived cell lines had a higher migratory capacity than the parental LM8 cell line (Figure 2F). In addition, the CTC-derived cell lines showed higher mRNA expression of VEGFa (Figure 2G).

Gene expression analysis further showed that the high CD44 expression of the LM8 cell line was maintained in the CTC-derived cell lines. IF stainings further confirmed the expression of CD44 on the surface of the CTC-derived cell lines (Figure 2H), which suggests that CD44 remains a stable feature of LM8-derived CTCs.

Clonogenic assays were performed after X-ray irradiation to identify further differences between the CTC-derived cell lines and the parental cell line with respect to resistance to radiotherapy. The plots of the surviving fraction showed no difference between the parental cell line and the CTC-derived cell lines (Supplementary Figure S1).

### 3.2. Transcriptomic Profiling Reveals Distinct Gene Expression Patterns in CTC-Derived Cell Lines

**Figure 3:**
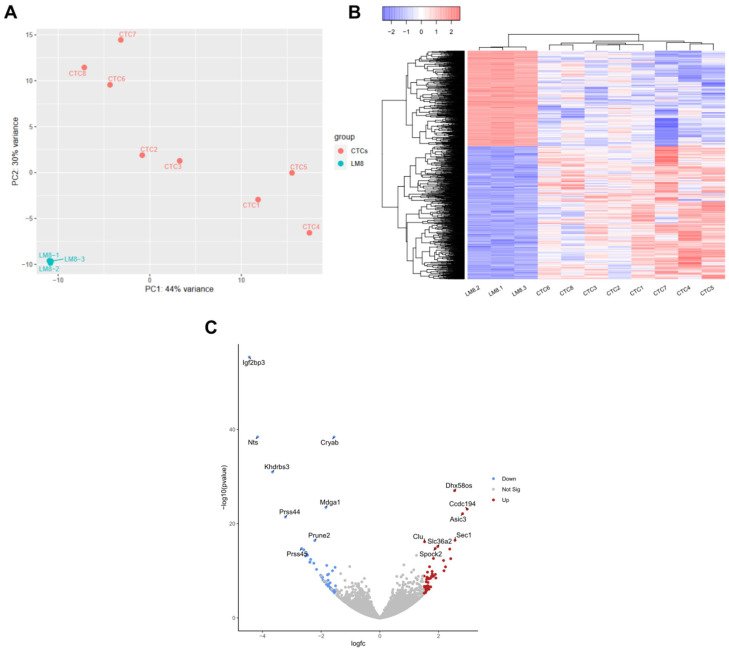
Gene expression comparison between parental and CTC-derived cell lines. (**A**) PCA plot showing all CTC-derived cell lines and three replicates of LM8, CTCs of 2 different experiments were analyzed. (**B**) Heatmap showing all DEGs (*p*-adj < 0.05) between the parental LM8 cell line and all CTC-derived cell lines, depicted are log2 (value + 1) transformed z-scores, complete Euclidean clustering. (**C**) Volcano plot showing DEGs between the parental cell line LM8 and CTC-derived cell lines, colors indicate a log(Fc) higher than 1.5 and a *p*-adj. < 0.05, 15 most differentially expressed Genes were labeled.

To further investigate the differences between parental and CTC-derived cell lines, we repeated the isolation of CTCs from LM8-bearing mice and included 3 additional CTC cell lines in an mRNA seq experiment. In this experiment, we then compared the gene expression of 8 CTC-derived cell lines with 3 parental LM8 cell lines. A principal component analysis (PCA) showed that the replicates of the LM8 cell line were tightly clustered, while the CTC-derived cell lines showed increased heterogeneity (Figure 3A). Furthermore, the PCA plot shows that the transcriptome of the CTC-derived cell lines differs substantially from that of the parental LM8 cell line. Since all cell lines were cultured under the same conditions, this indicates that the changes the CTCs underwent during their generation within the metastatic cascade were preserved during the isolation and cultivation process.

A differential expression analysis revealed 361 DEGs (log_2_(Fc) >1), as shown in Figure 3B. Among the most upregulated genes in the CTC-derived cells were Dhx58os, Ccdc194, Asic3, Sec1, Clu, Slc36a2, and Spock2. Conversely, the most downregulated genes in CTC cultures included Nts, Cryab, Khdrbs3, Mdga1, Prss44, Prune2, and Prss45 (Figure 3C). Interestingly, Igf2bp3 was the most downregulated gene in the CTC-derived cell lines, and its expression was almost fully suppressed (full list of DEGs in Supplementary Table S1).

### 3.3. Gene Ontology and Pathway Enrichment Analysis Reveal Distinct Molecular Signatures in CTC-Derived Cell Lines

**Figure 4:**
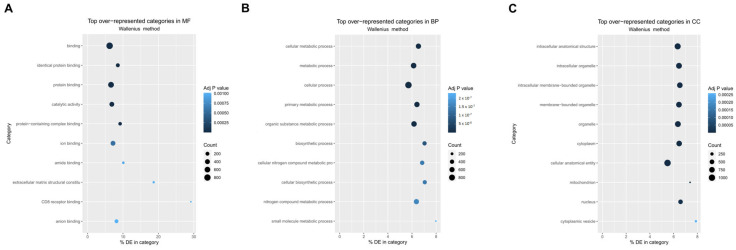
Gene expression comparison between parental and CTC-derived cell lines. (**A**) GO analyses of the DEGs between parental LM8 cell line and all CTC derived cell lines for the category Molecular Function (MF). (**B**) GO analyses of the DEGs between parental LM8 cell line and all CTC-derived cell lines for the category Biological Processes (BP). (**C**) GO analyses of the DEGs between parental LM8 cell line and all CTC derived cell lines for the category Cellular Compartments (CC).

Gene ontology (GO) analysis of the DEGs revealed that for Molecular Functions (MF), the most overrepresented categories were “Binding”, “Identical Protein Binding”, and “Protein Binding” (Figure 4A), and in terms of Biological Processes (BP), the enriched categories included “Cellular Metabolic Process”, “Metabolic Process”, and “Cellular Process” (Figure 4B). For Cellular Compartments (CC), the primary categories were “intracellular anatomical structure”, “intracellular organelle”, and “intracellular membrane-bounded organelle” (Figure 4C). As indicated by the principal component analysis, the gene expression of the CTC-derived cell lines differed significantly from the parental cell line. Therefore, the GO analysis of the DEGs revealed 452 overrepresented and 15 underrepresented GO terms (full list in Supplementary Table S2).

Among the enriched GO terms were cell death and apoptosis-related terms such as “negative regulation of programmed cell death” (GO: 0043069), “regulation of apoptotic signaling pathway” (GO: 2001233), and “apoptotic process” (GO: 0006915), indicating a difference in the regulation of cell death and apoptosis between the CTC-derived cell lines and the parental cell line.

**Figure 5:**
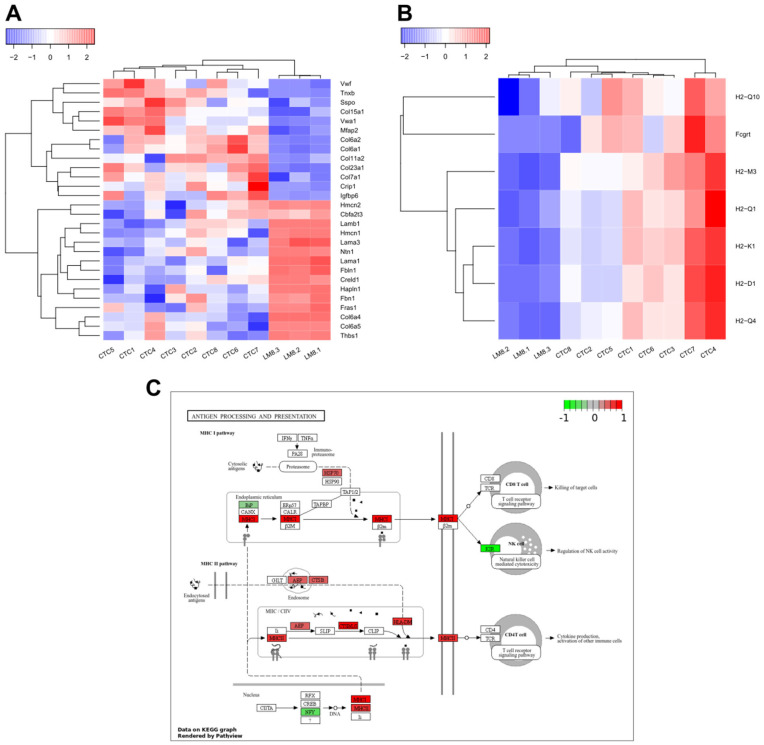
Gene expression comparison between parental and CTC-derived cell lines. (**A**) Heatmap of all DEGs in the MF GO term “Extracellular matrix structural constitution”. (**B**) Heatmap of all DEGs in the MF GO term “CD8 receptor binding”. (**C**) Visualization of the enriched KEGG pathway, mmu04612, “Antigen processing and presentation” with Pathview, red indicates increased expression in CTC-derived cell lines, red a decrease. Regular KEGG map notation applies, most important symbols: rectangles with black borders: gene products, solid arrow; molecular interaction or relation, dashed arrow; indirect link or unknown reaction, circles; chemical compounds, DNA, or other molecules.

Furthermore, extracellular matrix-related GO terms were enriched, like “extracellular matrix” (GO: 0031012), “collagen-containing extracellular matrix” (GO: 0062023), and “extracellular matrix structural constituent” (GO: 0005201). The DEGs contained in the GO term “extracellular matrix structural constituent” are visualized in Figure 5A. The downregulation of a few laminin-associated genes, such as Lamb1, Lama3, and Lama1, could be observed, while an upregulation of the collagen encoding genes Col6a2 and Col6a1 could be seen.

The GO analyses also revealed enrichment of the GO term “CD8 receptor binding” (GO: 0042610), which contains genes responsible for the formation of MHC1. The CTC-derived cell lines all expressed higher levels of genes associated with CD8 receptor binding (Figure 5B). Further KEGG analysis revealed major differences in “antigen processing and presentation” (mmu04612) between the parental and CTC-derived cell lines. It was observed that the CTC-derived cell lines had higher gene expression levels for a number of genes that play an important role in the processing and presentation of antigens via MHC1 and MHC2 (Figure 5C). This is particularly interesting because neither CTC-derived cell lines nor LM8 express Pdl1 or Ido1. In addition, in the context of MHC2 signaling, no CD80 or CD86 were expressed in CTCs and LM8. Another pathway enriched in the KEGG analysis was “phagosome” (mmu04145), where CTC-derived cell lines also expressed higher levels of vATPase and cathepsin-associated genes, indicating higher lysosomal activity (Supplementary Figure S2) (the full list of KEGG pathways in Supplementary Table S3).

## 4. Discussion

In this study, we successfully isolated, cultured, and characterized CTCs from a murine OS model. Accordingly, we were able to confirm the presence of viable and proliferating CTCs in the bloodstream of the animals, supporting the role of these cells in metastasis formation. Isolation and cultivation in our study were performed following the protocol of Tanaka et al. (2013) [19]. Tanaka et al. describe how cellular protrusions characteristic of the LM8 cell line are retained in LM8-CTC-derived cell lines. In our study, we were able to confirm their findings and found that not only the formation of protrusions was maintained (Figure 2C) but also the expression of several osteoblast-specific markers, such as RunX2, Osterix [30], and Osteocalcin [31] (Figure 2D). Since it was previously shown that lung metastasis of the LM8 cell line was reduced by irradiation [32,33], we investigated the radioresistance of the CTC-derived cell lines but found no difference compared to the parental LM8-cell line.

### 4.1. CD44 as a Marker for OS CTCs and Disease Progression

Frequent expression of CD44 has already been found in CTCs of epithelial origin in several PDX mouse models and in patients [10,34,35], while in our experiment, the retention of the same level of CD44 expression from primary tumor to the CTCs suggests that the CD44 status of the primary tumor can be inferred from the CD44 status of CTCs in OS (Figure 2H). Since CD44 may serve as a marker of progression in OS [36,37], monitoring CTCs for CD44 expression could serve as a non-invasive tool to monitor progression in OS patients. In addition, our findings showed that CD44 can be used as a CTC marker in the LM8/C3H mouse model to identify CTCs and differentiate them from PBMCs.

### 4.2. Adaptations of OS CTCs: Increased Migration, Nuclear Size Reduction, and VEGFa Expression

In agreement with other research groups [31,32], we found that our CTC-derived cell lines exhibited higher levels of migration than their parental cell line (Figure 2F). This effect has already been observed in CTC-derived cell lines from mesenchymal [38] and epithelial tumors [39]. The increased migration of the CTC-derived cell line in this study was most likely due to adaptations that the cells acquired in order to disseminate from the primary tumor and become CTCs. Our data suggest that one of these adaptations was a reduction in the size of the nucleus (Figure 2E). These findings are consistent with the published literature describing CTCs as being significantly smaller in nuclear size and total volume than cultured cell lines [40]. Since the nucleus is the largest and most rigid organelle in the cell [41], it is a limiting factor for cell migration through confined spaces. Therefore, reducing the size of the nucleus may facilitate the intravasation of CTCs across the endothelium into the blood vessels.

We were also able to show that the expression of VEGFa was increased in all CTC-derived cell lines compared to the paternal LM8 cell line (Figure 2G). As the expression of VEGFs is a prerequisite for the intravasation of CTCs across the endothelium [42] and is frequently found in CTCs of epithelial origin [43,44], our data is in line with the published literature and underscores the relevance of VEGFa expression in mesenchymal OS CTCs.

### 4.3. Gene Expression of LM8-CTCs Differ Significantly from Their Parental Cell Line

To uncover differences in gene expression and further adaptations of the parental cell line to release CTCs into the bloodstream, we compared the gene expression of 8 CTC-derived cell lines with their parental cell line LM8. The high level of heterogeneity in the CTC populations of epithelial origin has already been well described [45,46], and accordingly, we found a high level of heterogeneity between all CTC-derived cell lines, although their parental cell line was the same (Figure 3A).

Despite the high heterogeneity of CTCs, we identified 361 DEGs differentially expressed between CTC-derived cell lines and the parental LM8 cell line. Interestingly, Igf2bp3 was the most significantly downregulated gene in the CTC-derived cell lines (Figure 3C). Dai et al. (2023) [47] demonstrated, using in situ hybridization, that an increased number of CTCs expressing IMP3 (gene product of Igf2bp3) correlated with higher Enneking staging and was associated with the development of metastases in OS patients. Our mRNA sequencing data suggests that the downregulation of Igf2bp3 expression could be part of the metastatic cascade leading to the generation of CTCs in the LM8/C3H model.

A comparison of our DEGs with those identified by Green et al. (2020), who analyzed OS CTCs in patients compared to primary tumors [17], revealed several overlaps. Among the genes with statistically significant adjusted *p*-values (<0.05), the overlapping genes are mt-Nd2, Akt2, and Mmp14.

### 4.4. Immune Evasion Strategies of OS CTCs: Platelet Protection and Antigen Presentation

Once in the circulation, CTCs are exposed to the whole blood-borne immune system and, therefore, subjected to a high degree of immune surveillance and a plethora of immune cells. These include natural killer (NK) cells, which play an important role in the detection and elimination of CTCs [48]. Since NK cells eliminate cells that do not express sufficient MHC1 [49], there are several mechanisms by which CTCs can avoid NK cell-mediated lysis. One of these mechanisms is the acquisition of a protective shell consisting of platelets that protects the CTCs from direct contact with NK cells [50]. In our experiment, we observed an increased expression of Vwf in CTC-derived cell lines. Since Vwf is a potent inducer of platelet adhesion and aggregation [51], its increased expression in CTCs may lead to increased protection of CTCs from immune cells, which is necessary for their survival in the bloodstream.

Further GO analysis of all DEGs revealed that the CTC-derived cell lines expressed higher levels of MHC1- and MHC2-associated genes (Figure 5B). In addition, genes associated with antigen processing and presentation were significantly higher expressed in CTC-derived cell lines compared to the parental LM8 cell line (Figure 5C). The increased expression of MHC1 is likely to be another countermeasure of CTCs to avoid detection by NK cells. It has already been described how molecules can be exchanged between platelets and CTCs [52], and it has already been shown that platelets can deliver normal MHC1 proteins to the surface of cancer cells [53]. Upregulation of MHC1-associated genes would have the same effect in avoiding detection and elimination by NK cells. Therefore, the observed increase in MHC1 expression of CTCs is likely to be an addition to the immune evasion mechanisms mentioned above.

The increased expression of MHC2-associated genes that we observed in mesenchymal CTC-derived cell lines has also recently been observed in disseminated breast cancer cells. Lei et al. (2023) [54] found that breast cancer cells isolated from lymph node metastasis exhibited higher MHC2 expression while lacking costimulatory signals such as CD80 and CD86. In this study, we observed the same phenomenon in mesenchymal CTC-derived cell lines, expressing higher levels of MHC2 molecules while not expressing CD80 and CD86. As already suggested by Lei et al. for disseminated tumor cells, our data suggest that CTCs could promote immune tolerance through T cell anergy and thereby increase their ability to remain undetected by immune cells in the bloodstream.

### 4.5. Extracellular Matrix Remodeling in OS CTCs

The importance of ECM remodeling for tumor progression and CTC formation has already been thoroughly described in epithelial tumors [55]. Ting et al. (2014) showed major differences in the ECM composition between pancreatic CTCs and the corresponding primary tumor [12]. They identified, among others, Dcn, Sparc, Col1a2, and Col3a1 as significantly upregulated in CTCs compared to the primary tumor. In this study, we also found significant differences in ECM composition between OS CTCs and their corresponding primary cell line. We observed increased expression of several collagen genes, including Col6a1, Col6a2, and Col11a2, while several laminin genes were downregulated in CTC-derived cell lines (Figure 5A). The changes in the ECM, combined with the increased expression of VEGFa and the reduction of the nuclear size, may facilitate migration and extravasation. Accordingly, to this data, we observed a higher migratory capacity of the CTC-derived cell lines (Figure 2F).

Of particular interest is the upregulation of one gene in our CTC-derived cell lines that has previously been shown to play an important role in OS metastases, Col6a1 [56]. Zhang and his team showed that Col6a1 expression was significantly higher in the primary tumor of metastatic OS patients compared to non-metastatic patients. Furthermore, they showed that metastatic lesions showed increased expression of Col6a1 compared to primary OS tissue. They also showed that Col6a1 plays an important role in the migration and metastases formation of several OS cell lines. The increased expression we have now observed in CTC-derived cell lines further highlights its role in metastasis formation and suggests that an increase in Col6a1 leads to a higher number of metastases by promoting the formation of CTCs.

## 5. Conclusions

In this study, we have successfully isolated, cultivated in vitro, and characterized CTCs from a murine osteosarcoma model, and we confirmed the role of this aggressive subpopulation of cells in metastasis formation. The CTC-derived cell lines retained key osteosarcoma markers and showed increased migration and significant transcriptomic differences from the parental LM8 cell line. We were able to identify genes that are linked to antigen presentation and extracellular matrix remodeling. These genes were differently expressed, suggesting an adaptation that enhances CTC survival into the bloodstream and metastatic capability. We believe that our findings could provide valuable insights into osteosarcoma CTC biology and may guide future therapeutic strategies targeting metastatic progression.

## Data Availability

The original data presented in the study are submitted to SRA under the bioproject PRJNA1238064.

## Supplementary Materials

The following supporting information can be downloaded at: https://www.mdpi.com/article/10.3390/cancers17071210/s1, Figure S1: Clonogenic survival after irradiation for LM8 and CTC derived cell lines; Figure S2: Visualized KEGG pathway “Phagosome”; Table S1: All DEGs; Table S2: All Gos; Table S3: KEGG pathways.

## Abbreviations

The following abbreviations are used in this manuscript:

BP	Biological processes
BSA	Bovine serum albumin
CC	Cellular components
CD	Cluster of differentiation
CTCs	Circulating tumor cells
DEGs	Differentially expressed genes
DMEM	Dulbeccos Modified Eagle Medium
DTCs	Disseminated tumor cells
ECM	Extracellular matrix
EMT	Epithelial to mesenchymal transition
FBS	Fetal bovine serum
GO	Gene ontology
IF	Immunofluorescence
KEGG	Kyoto Encyclopedia of Genes and Genomes
MF	Molecular Functions
MHC	Major histocompatibility complex
MTA1	Metastasis associated protein 1
NK cells	Natural Killer cells
OS	Osteosarcoma
PBMCs	Peripheral blood mononuclear cell
PBS	Phosphate-buffered saline
PCA	Principle component analysis
PCR	Polymerase chain reaction
Pdl1	Programmed death-ligand 1
RBCs	Red blood cells
SRY	Sex determining region of Y
VEGFa	Vascular endothelial growth factor A
WBCs	White blood cells
