# Supplementary material for: Gene Expression Profiling and Phenotypic Characterization of Circulating Tumor Cells Derived from a Murine Osteosarcoma Model
Source: Cancers (Basel). 2025 Apr 2;17(7):1210. doi: 10.3390/cancers17071210 (PMC11988136; doi:10.3390/cancers17071210)
Supplement: Supplementary file 1 [file cancers-17-01210-s001.zip › Supplementary Figures.pdf]

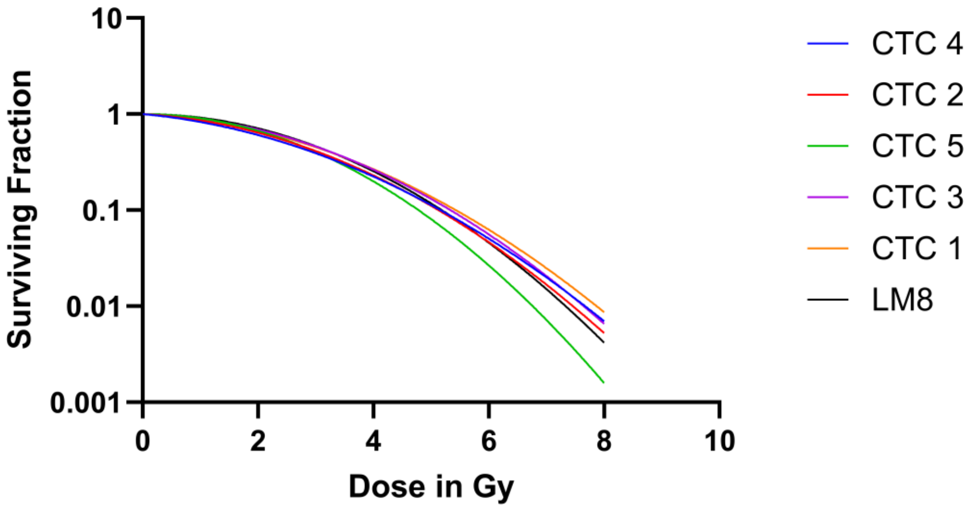

Supplementary Figure S1: Clonogenic survival after irradiation for LM8 and CTC derived cell lines

LM8 and CTC derived cell lines were irradiated as described in the materials and methods and the surviving fraction was plotted against the received dose in Gy. The doses were 0, 2, 4, 6, 8 Gy and data points were fitted in Graphpad Prism to the linear quadratic model.

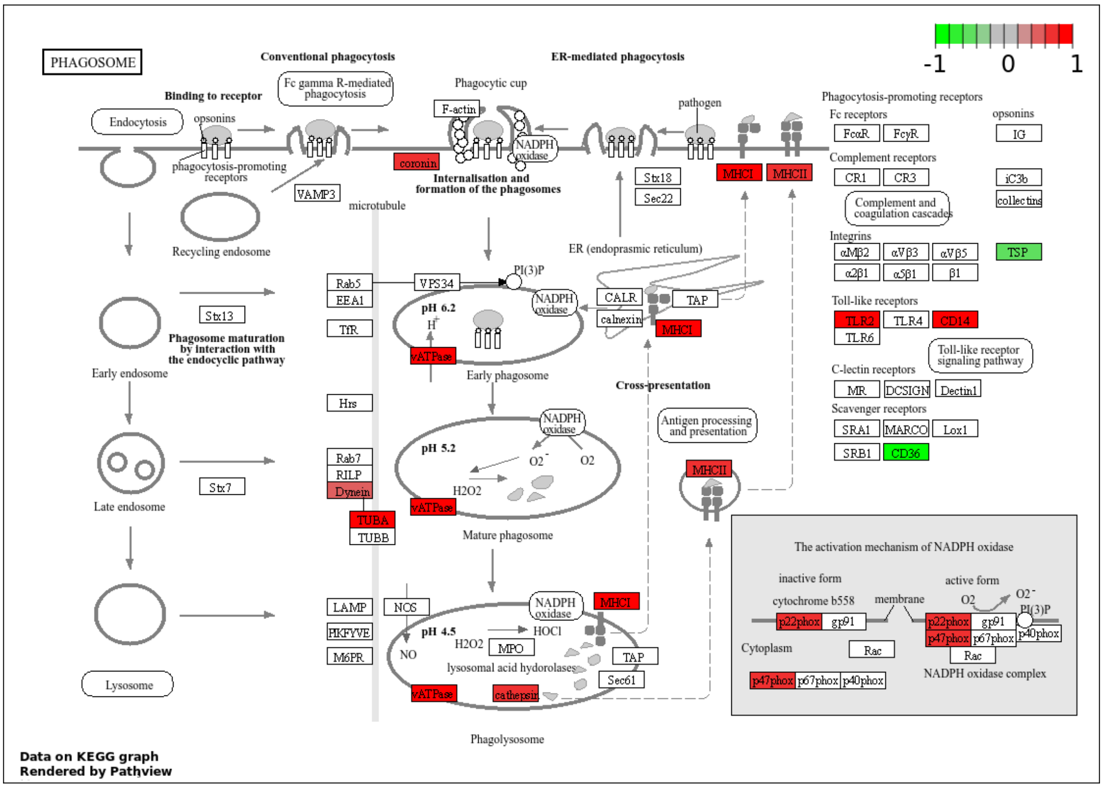

Supplementary Figure S2: Visualized KEGG pathway “Phagosome”

KEGG pathway „Phagosome“ differed significantly between LM8 and CTC-derived cell lines and was rendered by Pathview
